# Fecal pancreatic elastase-1 levels in older individuals without known gastrointestinal diseases or diabetes mellitus

**DOI:** 10.1186/1471-2318-11-4

**Published:** 2011-01-25

**Authors:** Karl-Heinz Herzig, Anna-Kaisa Purhonen, Kati M Räsänen, Joanna Idziak, Petri Juvonen, Ryszard Phillps, Jaroslaw Walkowiak

**Affiliations:** 1 Department of Biotechnology and Molecular Medicine, A.I. Virtanen Institute for Molecular Sciences, University of Eastern Finland, Kuopio, Finland; 2 Institute of Biomedicine, Department of Physiology, University of Oulu, Oulu, Finland; 3 Biocenter Oulu, University of Oulu, Oulu, Finland; 4 Department of Surgery, Kuopio University Hospital, Kuopio, Finland; 5 Department of Ophthalmology, District Hospital, Poznan, Poland; 6 I. Chair of Pediatrics, Department of Gastroenterology & Metabolism, Poznan University of Medical Sciences, Poznan, Poland

## Abstract

**Background:**

Structural changes occur in the pancreas as a part of the natural aging process. With aging, also the incidence of maldigestive symptoms and malnutrition increases, raising the possibility that these might be caused at least in part by inadequate pancreatic enzyme secretion due to degenerative processes and damage of the gland. Fecal elastase-1 is a good marker of pancreatic exocrine secretion. The aim of this study was to investigate the fecal elastase-1 levels among over 60 years old Finnish and Polish healthy individuals without any special diet, known gastrointestinal disease, surgery or diabetes mellitus.

**Methods:**

A total of 159 patients participated in this cross-sectional study. 106 older individuals (aged 60-92 years) were recruited from outpatient clinics and elderly homes. They were divided to three age groups: 60-69 years old (n = 31); 70-79 years old (n = 38) and over 80 years old (n = 37). 53 young subjects (20-28 years old) were investigated as controls. Inclusion criteria were age over 60 years, normal status and competence. Exclusion criteria were any special diet, diabetes mellitus, any known gastrointestinal disease or prior gastrointestinal surgery. Fecal elastase-1 concentration was measured from stool samples with an ELISA that uses two monoclonal antibodies against different epitopes of human elastase-1.

**Results:**

Fecal elastase-1 concentrations correlated negatively with age (Pearson r = -0,3531, *P *< 0.001) and were significantly lower among subjects over 70 years old compared to controls (controls vs. 70-79 years old and controls vs. over 80 years old, both *P *< 0.001). Among the over 60 years old subjects, the fecal elastase-1 concentrations were below the cut off level of 200 μg/g in 23 of 106 (21.7%) individuals [mean 112 (86-138) μg/g] indicating pancreatic exocrine insufficiency. Of those, 9 subjects had fecal elastase-1 level below 100 μg/g as a marker of severe pancreatic insufficiency.

**Conclusion:**

In our study one fifth of healthy older individuals without any gastrointestinal disorder, surgery or diabetes mellitus suffer from pancreatic exocrine insufficiency and might benefit from enzyme supplementation therapy.

## Background

The structural changes occurring in the pancreas as a part of the natural aging process include patchy fibrosis, lipomatosis, ductal epithelial hyperplasia, ductal widening and intraluminal protein deposition which have been documented in postmortem studies [1-4]. With aging, also the incidence of maldigestive symptoms and malnutrition increases, raising the possibility that these might be caused at least in part by inadequate pancreatic enzyme secretion due to degenerative processes and damage of the gland.

Only very few previous studies have addressed the effect of aging on pancreatic exocrine secretion capacity and those have reported inconsistent results. Gullo et al did not find any decrease in exocrine pancreatic function measured by pancreolauryl test in older people (aged 66 to 88 years, mean age 78) [5] nor when investigated by fecal elastase-1 tests in over 90 years old subjects [6]. In contrast, other studies demonstrated that lipase, chymotrypsin and bicarbonate secretion after secretin and cholecystokinin (CCK) stimulation decreases with age [7] and approximately 40% reduction in enzyme output was observed in older people under secretin and cerulein stimulation as compared with the younger subjects [8]. In an older population with a mean age of 58.7 years significantly lower secretion rates for bicarbonate following secretin and for the enzymes lipase and amylase following pancreozymin administration compared to a healthy young population with a mean age of 31.8 years have been reported [9]. A large-scale, population based, cohort study evaluated pancreatic exocrine secretion in 50 to 75 years old subjects [10]. In this cohort 11.5% showed signs of exocrine pancreatic insufficiency (elastase level < 200 μg/g stool) and 5.1% severe insufficiency (elastase level < 100 μg/g stool). 11.4% of the study population had a history of diabetes mellitus [10]. These studies suggest that pancreatic enzyme secretion may be reduced even in normal, healthy older people with no gastrointestinal diseases due to atrophy, fibrosis, sclerosis and lipomatosis of the organ.

Direct tests like the secretin-CCK or secretin-caerulein test have the highest sensitivity and specificity for the detection of exocrine pancreatic insufficiency, and they remain the gold standard for testing pancreatic exocrine function but they have various practical disadvantages being time consuming, invasive and expensive. Since pancreatic elastase-1 is very stable during intestinal transit and is enriched five- to six fold in feces, it can be used in the assessment of the pancreatic exocrine secretory capacity [11,12]. Measuring fecal elastase-1 concentration correlates moderately with direct tests [13] and has several advantages being non-invasive, inexpensive and allowing sample storage in room temperature up to five days. Many comparative studies support the value of fecal elastase-1 test in the assessment of the pancreatic secretion capability. In addition, being species specific enzyme, human elastase-1 can be differentiated from the porcine elastase present in enzyme supplements.

The aim of our study was to evaluate the pancreatic exocrine secretion in older people with no known gastrointestinal disease, surgery or diabetes mellitus with a simple fecal elastase-1 test to estimate the possible need for exogenous pancreatic enzyme replacement therapy in the older population.

## Methods

### Subjects

The study subjects were competent healthy individuals who were recruited from local elderly homes and health care centers from Eastern Finland and the ophthalmology outpatient clinic at the Department of Ophthalmology, District Hospital, Poznan, Poland. The subjects signed an informed consent and the study was accepted by the ethical committee of Hospital district of Northern Savo, Kuopio, Finland and the ethical committee of Poznan University of Medical Sciences, Poland. All procedures complied with the Helsinki Declaration.

The inclusion criteria were over 60 years of age, normal status and competence. Exclusion criteria were any special diet, alcohol abusers, diabetes mellitus, any known gastrointestinal disease or prior gastrointestinal surgery. None of the subjects had been diagnosed with exocrine pancreatic insufficiency or had been treated with pancreatic enzymes before the study.

A total of 159 subjects participated in the study. Older individuals (total n = 106) were divided into three age groups i) 60-69 years old (n = 31), ii) 70-79 years old (n = 38), and iii) over 80 years old (n = 37). 53 young controls (age 20-28 years) served as the control group. The same selection criteria were applied to controls as well as to older subjects.

The fecal elastase-1 was measured from 1 g of stool by an enzyme-linked immunosorbent assay (ScheBo^® ^Tech, Giessen, Germany). The test uses two monoclonal antibodies against different epitopes of human elastase-1. The intra- and inter-assay variations were 5.8% and 7.7%, respectively. Each sample was measured in duplicates. To minimize the intra-individual variation, the measurement was repeated from three stool samples from each subject and the average of the three samples was used for calculation of the results. The cut-off level for pancreatic insufficiency is elastase-1 concentration below 200 μg/g stool and for severe pancreatic insufficiency below 100 μg/g stool.

#### Statistical analysis

Normal distribution of the data was tested by Kolmogorov-Smirnov test. To compare the elastase-1 levels between the groups, one-way ANOVA with Tukey's multiple comparison test as post test was used. *P *< 0.05 was considered statistically significant. Correlation was tested by Pearson test. GraphPad Prism was used for statistical analysis (GraphPad Software Inc., San Diego, CA).

## Results

**Figure 1:**
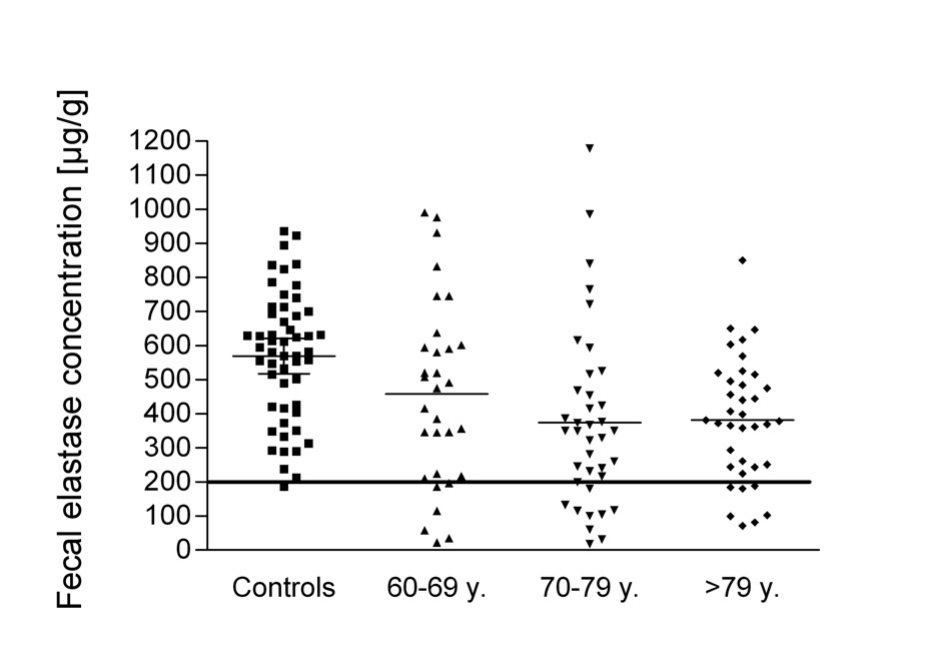
**Fecal elastase-1 concentration of subjects in different age groups with mean values indicated by lines**. Controls have significantly higher elastase-1 levels compared to 70-79 years old or over 80 years old subjects. The cut off level for pancreatic exocrine insufficiency of 200 μg/g is marked by a solid line.

**Figure 2:**
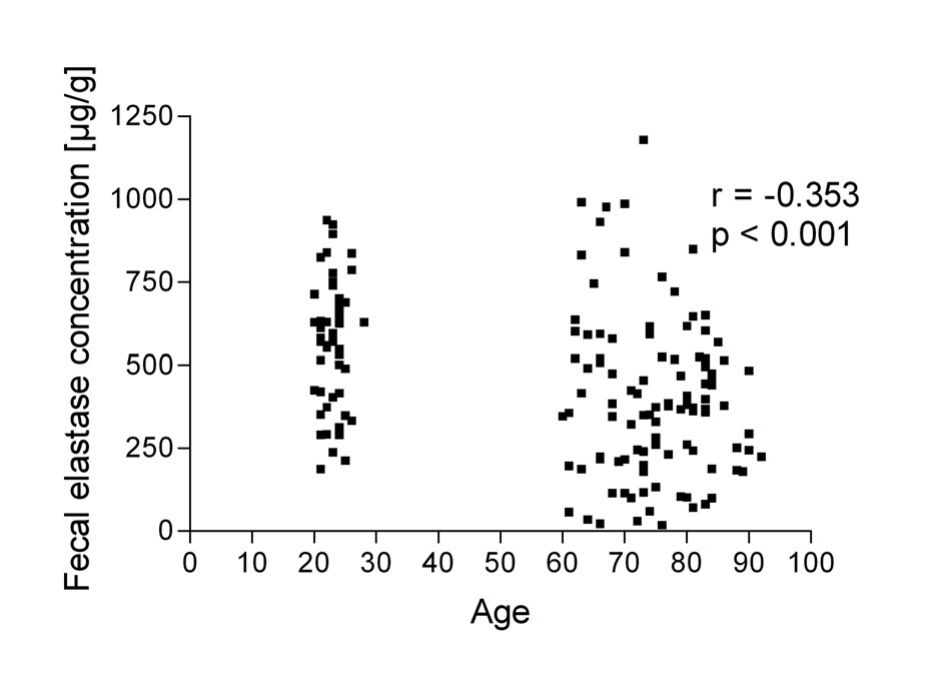
**Fecal elastase-1 level correlates negatively with age**. Pearson correlation r = - 0.353, P < 0.001.

Fecal elastase-1 concentration was (means and 95% confidence intervals are given) 570 (518-621) μg/g in controls, 458 (358-558)μg/g in 60-69 years old, 375 (289-461) μg/g in 70-79 years old and 381 (321-442) μg/g in over 80 years old subjects (Figure 1). The fecal elastase-1 concentration was significantly lower in individuals over 70 years old compared to controls (controls vs. 70-79 years and controls vs. over 80 years, both *P *< 0.001). The difference between controls and 60-69 years old and the differences between the older age groups were not statistically significant (*P *> 0.05). Fecal elastase-1 concentration correlated negatively with age (Pearson r = -0,3531, *P *< 0.001, Figure 2). Among the subjects over 60 years old, the fecal elastase-1 concentration was below the cut off level of 200 μg/g in 23 of 106 (21.7%) individuals [mean 112 (86-138) μg/g] indicating pancreatic exocrine insufficiency. 9 subjects had fecal elastase-1 level below 100 μg/g indicating severe pancreatic insufficiency. In the control group, one subject had an elastase-1 level below 200 μg/g.

## Discussion

In our investigation we found that 21.7% of subjects aged over 60 years without any known gastrointestinal diseases, gastrointestinal surgery or diabetes mellitus had fecal elastase-1 levels below 200 μg/g indicating pancreatic exocrine insufficiency. Reduction in exocrine secretion capacity leads to maldigestion of nutrients which might manifest as clinical symptoms like malnutrition, steatorrhoea, diarrhea, abdominal pain and weight loss [14]. In addition, as the cause of abdominal pain remains unclear in about 30% of the cases, pancreatic insufficiency should be considered as a possible etiology in these situations. Our results are comparable with other studies. Rothenbacher and others included also diabetics in their large population based study and found exocrine insufficiency (moderate or severe) in 22.7% and 19% of 65-69 years and 70-75 years old subjects, respectively [10]. Another study reported elastase-1 levels below 200 μg/g in 18.1% of the nondiabetic control subjects (mean age 58 years, range 22-80 years) [15]. In contrast, Gullo et al found that only one of 68 subjects over 90 years old and none in their control group (mean age 52 years, range 21-81 years) had low fecal elastase-1 levels [6] and there was no correlation between fecal elastase-1 and age. The discrepancy might be explained by tight selection criteria applied by Gullo and coworkers as their inclusion criteria included normal nutritional status and lifetime absence of important diseases, including digestive diseases, diabetes mellitus and alcohol abuse, suggested weight for height and normal biochemical examinations of several parameters possibly leading to exclusion of the cases with pancreatic insufficiency.

Measuring fecal elastase-1 level is the most appropriate indirect test to evaluate pancreatic exocrine function currently available at the clinics. The sensitivity of fecal elastase-1 measurement in detecting severe exocrine insufficiency is excellent (100% in [13,16,17] and 82% in [18]). In contrast, its sensitivity in mild pancreatic insufficiency in different studies has been reported to be ranging from 16,7 to 65% [13,16-20]. Some of the studies have been criticized for their small sample size or for underestimation of pancreatic insufficiency. However, considering the modest sensitivity of the test in detecting mild exocrine insufficiency, it should be noted that the actual prevalence of mild reduction in exocrine secretion capacity among older people is expected to be even higher than indicated by fecal elastase-1 measurements. It is well known that the incidence of disturbances in the endocrine pancreatic function increases with age [21] yet the deterioration of the exocrine gland has gained much less attention. Interestingly, several studies have reported higher prevalence of impaired exocrine secretion capacity among diabetic patients than in nondiabetic controls [15,22-24] although the association has not been confirmed by all [10]. Different theories have been proposed why diabetes could impair exocrine secretion including atrophy of the exocrine tissue due to lack of trophic insulin, fibrosis as a result of angiopathy, impaired exocrine regulation due to neuropathy or vice versa suggesting that primary exocrine disease might cause diabetes mellitus [15]. As the pathophysiology of these events remains unknown, morphological studies support the connection between exocrine and endocrine disease since changes of the pancreas were observed among diabetics, among those with exocrine insufficiency and increasingly so in those patients with both disorders [25]. In addition to other pathophysiological mechanisms, the natural process of aging and degeneration could affect the endocrine as well as the exocrine part.

In our study, we evaluated fecal elastase-1 levels in patients without known diabetes mellitus. The total prevalence of diabetes is between 10-20% among 60-79 year old people and approximately half of these cases are undiagnosed [21]. Thus it is likely that our study included also some subjects with undiagnosed diabetes since we did not screen them by laboratory tests. The most common causes of exocrine pancreatic insufficiency are chronic pancreatitis and pancreatic surgery followed by other diseases like cystic fibrosis, acute necrotizing pancreatitis, Shwachman Diamond syndrome, celiac disease, irritable bowel syndrome and inflammatory bowel disease [26-29]. Patients with any gastrointestinal surgery or known gastrointestinal disease were excluded from our study but besides the impact of aging, also underlying undiagnosed diseases remain as possible etiological factors for reduced fecal elastase-1 levels. Nevertheless, our study suggests that among the apparently healthy older subjects without any known gastrointestinal disease there is a considerable portion of subjects who might benefit from pancreatic enzyme supplementation therapy. The decline of fecal elastase-1 level below the cut off level 200 μg/g may indicate already atrophy and degeneration of the gland. However, no correlation was found between fecal elastase-1 levels and clinical symptoms of abdominal discomfort [15]. Clinically relevant maldigestion may occur earlier than the appearance of overt symptoms [30]. According to present recommendation, every patient with exocrine pancreatic insufficiency should be treated with pancreatic enzyme replacement therapy regardless of the amount of steatorrhoea, maldigestion or clinical symptoms [31].

## Conclusions

Among older individuals without any known gastrointestinal disease, surgery or diabetes mellitus, one fifth of the individuals had abnormal fecal elastase-1 levels indicating pancreatic exocrine insufficiency. In the general unselected population with a high incidence of diabetes mellitus, malabsorption syndromes and abdominal surgery the incidence might be even higher. It is important to increase the awareness of these problems in this population group among general physicians since for these patients, careful clinical examination and possibly pancreatic enzyme replacement therapy are warranted.

## Competing interests

The authors declare that they have no competing interests.

## Authors' contributions

KHH, AKP and JW: study concept and design, acquisition of subjects, interpretation of data, preparation of manuscript. KR, JI: analysis of samples, interpretation of data, manuscript preparation. KR, PJ, RP: acquisition of subjects, manuscript preparation. All authors read and approved the final manuscript.

## Pre-publication history

The pre-publication history for this paper can be accessed here:

http://www.biomedcentral.com/1471-2318/11/4/prepub
